# Geostatistical modelling of the association between malaria and child growth in Africa

**DOI:** 10.1186/s12942-018-0127-y

**Published:** 2018-02-27

**Authors:** Benjamin Amoah, Emanuele Giorgi, Daniel J. Heyes, Stef van Burren, Peter John Diggle

**Affiliations:** 1 CHICAS Research Group, Lancaster Medical School, Lancaster University, Bailrigg, Lancaster, UK; 2 Liverpool School of Tropical Medicine, Pembroke Place, Liverpool, UK; 3 Department of Child Health, Netherlands Organization for Applied Scientific Research TNO, Leiden, The Netherlands; 4 Department of Methodology and Statistics, Utrecht University, Utrecht, The Netherlands

**Keywords:** Child growth, Exceedance probability, Geostatistics, Malaria, Stunting

## Abstract

**Background:**

Undernutrition among children under 5 years of age continues to be a public health challenge in many low- and middle-income countries and can lead to growth stunting. Infectious diseases may also affect child growth, however their actual impact on the latter can be difficult to quantify. In this paper, we analyse data from 20 Demographic and Health Surveys (DHS) conducted in 13 African countries to investigate the relationship between malaria and stunting. Our objective is to make inference on the association between malaria incidence during the first year of life and height-for-age Z-scores (HAZs).

**Methods:**

We develop a geostatistical model for HAZs as a function of both measured and unmeasured child-specific and spatial risk factors. We visualize stunting risk in each of the 20 analysed surveys by mapping the predictive probability that HAZ is below − 2. Finally, we carry out a meta-analysis by modelling the estimated effects of malaria incidence on HAZ from each DHS as a linear regression on national development indicators from the World Bank.

**Results:**

A non-spatial univariate linear regression of HAZ on malaria incidence showed a negative association in 18 out of 20 surveys. However, after adjusting for spatial risk factors and controlling for confounding effects, we found a weaker association between HAZ and malaria, with a mix of positive and negative estimates, of which 3 out of 20 are significantly different from zero at the conventional 5% level. The meta-analysis showed that this variation in the estimated effect of malaria incidence on HAZ is significantly associated with the amount of arable land.

**Conclusion:**

Confounding effects on the association between malaria and stunting vary both by country and over time. Geostatistical analysis provides a useful framework that allows to account for unmeasured spatial confounders. Establishing whether the association between malaria and stunting is causal would require longitudinal follow-up data on individual children.

**Electronic supplementary material:**

The online version of this article (10.1186/s12942-018-0127-y) contains supplementary material, which is available to authorized users.

## Background

Undernutrition underlies 45% of all child deaths among children under 5 years [1]. A very low height-for-age, usually referred to as stunting, is an important indicator that reflects the cumulative effects of undernutrition and disease infections [2]. Stunted children are more prone to illness and premature death. Stunting among children is known to be associated with poor cognitive development [3, 4]. Long-term consequences of stunting include lower adult economic productivity, higher risks of ill-health and, among women with short stature, an increased risk of death during delivery [5–8]. Globally, the rate of stunting in children under 5 years reduced from 32.7% (198 million) in year 2000 to 23.2% (156 million) in year 2015 [9]. In Africa however, the rates reduced from 38% in 2000 to 32% in 2015, representing more limited progress than in Asia, Latin America and the Caribbean where stunting rates dropped by more than one third over the same period [9]. In many low- and middle-income countries (LMICs), over 50% of 12–23 months old children are stunted [10–12]. In 2014, less than half of all children under 5 years lived in LMICs, yet these countries accounted for two-thirds of all stunted children globally [13]. Although the main risk factor for stunting is inadequate nutrition, exposure to infectious diseases may also lead to an increase in stunting risk [14, 15]. However, there are indirect effects of malaria not fully understood [16, 17], and it is unclear if part of the stunting burden can be attributed to malaria.

Malaria is still a public health threat, although the ongoing global fight against it has resulted in 50% decrease in the infection prevalence and 40% decrease in the clinical incidence in the endemic region of Africa between 2000 and 2015 [18]. In 2015, there were an estimated 214 million malaria cases and 438 thousand deaths from malaria worldwide, of which 88% occurred in sub-Saharan Africa and 70% in children under the age of 5 years, with 10% of all deaths in children under the age of 5 years due to malaria [19]. In 2017, similar global estimates were reported: 216 million malaria cases and 445 thousand malaria deaths, of which 91% occurred in sub-Saharan Africa, with most of the deaths still occuring in children under 5 years [20]. The association between malaria and stunting is unclear and still a matter of debate, with studies showing contrasting results. For example, maternal malaria has been found to impact on child growth [21], with infants born to women who experienced malaria during pregnancy having an increased risk of impaired height and weight gain [22–25]. The risk of stunting has been found to increase for every malaria episode [26]. On the other hand, some studies suggest that stunting may modulate susceptibility to malaria, especially during the first 2 years of life [27, 28]. Whilst some studies suggest that stunted children may be at higher risk of developing malaria episodes [29], others report that stunting may have a protective effect against malaria [30, 31]. In other studies, instead, no association is found [32, 33]. More recently, Fink et al. [34] found a significant effect of malaria exposure on cognitive development and socio-emotional development, but not on height, for which they report an estimated effect of about 3.000 and associated 95% confidence interval (− 11.350, 4.606).

The height-for-age Z-score (HAZ) measures the deviation from heights based on the World Health Organization (WHO) growth standards [35, 36] and are comparable across ages and gender. Values of HAZ below − 2 are used as an indicator of stunted growth. In this paper, we analyse data from 20 Demographic and Health Surveys (DHS) conducted in Senegal, Mozambique, Ghana, Burkina Faso, Zambia, Malawi, Rwanda, Cote d’Ivoire, Burundi, Liberia, Namibia, Togo and Tanzania to pursue the following objectives: (1) to investigate the association between malaria and HAZ by developing a geostatistical framework that accounts for both measured and unmeasured risk factors for stunting; (2) to understand how such association varies across the African countries considered in this study; (3) to map the risk of stunting. We also discuss the limitations of this study and provide a detailed description on how the proposed modelling framework could be further extended to a longitudinal setting. To the best of our knowledge, this is the first study that investigates the association between the geographical distribution of malaria and HAZ using a model-based geostatistical approach.

## Methods

### Data

DHS are nationally representative household surveys that are generally repeated every 5 years and provide information on a range of health and population indicators, including anthropometric information. The DHS methodology is usually based on a stratified two-stage cluster design. At the first stage, enumeration areas are drawn from census files. At the second stage, for each enumeration area selected, samples of households are drawn from an updated list of households to form groups of households known as sampling clusters. The GPS location of the center of each sampling cluster is taken as the cluster location. Each child is allocated to a spatially-referenced sampling cluster. We analyse data from 20 DHS conducted between 2003 and 2014 [37]. Table 1 shows the number of clusters and individuals for each survey. The average number of children per cluster varies from one survey to another, with the highest value of about 21.7 in Burkia Faso in 2003 and the lowest of about 5.7 in Malawi in 2010.

**Table 1:** Sample size summaries for the analysed DHS data indicating the country, year of survey, number of children, number of sampled clusters, and average number of children per cluster

Country	Year	No. of children	No. of clusters	Average no. of children per cluster
Senegal	2005	2710	355	7.6
Senegal	2011	3694	384	9.6
Mozambique	2011	9595	609	15.8
Ghana	2003	3010	393	7.7
Ghana	2008	2350	393	6.0
Ghana	2014	2671	410	6.5
Burkina Faso	2003	8581	396	21.7
Burkina Faso	2010	6290	540	11.6
Zambia	2007	5243	317	16.5
Zambia	2014	4635	303	15.3
Malawi	2004	6238	386	16.2
Malawi	2010	4623	811	5.7
Rwanda	2005	3692	455	8.1
Cote d’Ivoire	2007	3305	288	11.5
Burundi	2010	3449	376	9.2
Liberia	2007	4197	270	15.5
Liberia	2013	3206	319	10.1
Namibia	2007	3669	484	7.6
Togo	2014	3209	328	9.8
Tanzania	2010	6581	453	14.5

The variables used in the analysis are the following.

*Child-specific variables* Data on a child’s height, age and gender, family’s wealth index and mother’s education level were obtained from the DHS for all sampled children aged less than 5 years. Families’ wealth indices are constructed using principal component analysis on household’s ownership of television, radio, watch, vehicles and agricultural land, type and number of animals owned, bank account, materials used for housing construction, type of water access and sanitation facilities [38].

*Urban extent indicator* We use information on urban extents, available as raster data at a spatial resolution of 1 km by 1 km, from the Global Rural-Urban Mapping Project [39]. This variable is a binary indicator that classifies each spatial grid cell as urban or rural, based on a combination of population counts, settlement points, and presence of night-time lights.

*Estimated malaria incidence rates* We use raster data on estimated *Plasmodium falciparum* incidence as obtained from a Bayesian spatio-temporal model implemented by the Malaria Atlas Project [18]. The data are available at a temporal resolution of 1 year, from 2000 to 2015, and a spatial resolution of 0.05° × 0.05°. More specifically, the estimated *Plasmodium falciparum* malaria incidence at pixel-level is the predicted average clinical incidence rate per child per year in the age cohorts 0–5 years. A clinical malaria episode is an attributable febrile episode with a body temperature in excess of 37.5 °C. Multiple bouts of symptoms occurring within a 30-day period are counted as a single episode.

### Model formulation and spatial prediction

Accounting for spatial effects is crucial in order to deliver valid inferences on the regression coefficients [40]. Model-based geostatistics allows us to incorporate both explained and unexplained (residual) spatial variation in HAZ and to predict the risk of stunting throughout a geographical area of interest.

Let $Y_{ij}$ denote the HAZ for the *j* *th* sampled child at the cluster location $x_{i}$. We distinguish between two sources of variation in HAZ: between-cluster variation, induced by spatially varying risk factors; and within-cluster variation due to child-specific characteristics. Each of these components depends on both measured and unmeasured risk factors. In order to account for the latter, we define a hierarchical linear model as follows. Let $S(x_{i})$ denote a stationary Gaussian process and $U_{i}$ represent mutually independent zero-mean Gaussian variables with common variance $\tau ^2$. We assume that, conditionally on $S(x_{i})$ and $U_i$, the $Y_{ij}$ are Gaussian variables with means $\mu _{j}(x_{i})$ and variance $\omega ^2$, where

1 $$\begin{aligned} \mu _{j}(x_i) = e_{ij}^\top \gamma + d(x_i) \beta + \delta {\mathcal {M}}_{ij} + f({\mathcal {A}}_{ij}) + U_i + S(x_i),\,{\text {for}}\,&\,i=1,\ldots ,n\nonumber \\&j=1,\ldots , m_{i}. \end{aligned}$$

In (1), *n* is the number of cluster locations and $m_i$ is the number of individuals at cluster location $x_i$. In (1) we also distinguish between three types of explanatory variables: $e_{ij}$, a vector of child-specific explanatory variables, including sex, family’s wealth index and mother’s education level; $d(x_i)$, a spatial indicator variable which takes values 1, if location $x_{i}$ is classified as urban and 0 if rural; ${\mathcal {M}}_{ij}$, the estimated malaria incidence at location $x_{i}$ during the first year of life of the *j*-th child. The parameters $\gamma$, $\beta$ and $\delta$ are the regression parameters associated with each of the three types of explanatory variables, whilst $f({\mathcal {A}})$ is a cubic spline function of age, $\mathcal {A}$, with knots at 12 and 24 months.

Our objective is to make inference on the parameter $\delta$, which quantifies the effect of malaria incidence in the first year of life on HAZ. Our assumption is that malaria has a lagged effect on height and, therefore, we use the incidence of malaria during the first year of life to determine the strength of this association. In the remainder of the paper, we shall refer to the parameter $\delta$ and the variable ${\mathcal {M}}_{ij}$ in (1) as the effect of malaria on HAZ and malaria incidence, respectively.

In (1), the unstructured random effect $U_i$ conflates two sources of residual variation: spatial variation on a scale smaller than the minimum observed distance between clusters; and unexplained unstructured variation at cluster level.

The spatially structured residuals *S*(*x*) are modelled as a zero-mean stationary and isotropic Gaussian process with variance $\sigma ^2$ and exponential correlation function given by

2 $$\begin{aligned} \rho (u; \phi ) = \exp (-u/\phi ), \end{aligned}$$

where *u* is the Euclidean distance between any two locations. The scale parameter $\phi$ regulates the rate at which the spatial correlation decays with increasing distance *u*.

We map the risk of stunting for male children, 24 months old, using the predictive probability that HAZ is below − 2 over a 0.05° × 0.05° grid. We integrate out the effect of maternal education and wealth index using the following Monte Carlo approach. We generate 10,000 samples from the joint distribution of these two variables and, conditionally on these, we then simulate values of HAZ. The stunting risk is then computed by taking the proportion of simulated HAZ samples that are below − 2.

More details on the computational implementation and on the mapping of stunting risk are given in Additional file 1.

### Model validation

To check the validity of the adopted spatial correlation structure for the data, we carry out the following Monte Carlo procedure. We simulate 1000 empirical variograms under the fitted model and then use these to compute 95% confidence intervals at any given spatial distance of the variogram. If the empirical variogram obtained from the data falls within the 95% tolerance bandwidth, we conclude that the adopted spatial correlation function is compatible with the data. If, instead, that falls outside the 95% tolerance bandwidth, then the data show evidence against the fitted model. More details are provided in Additional file 1.

### Understanding the variation in the effect of malaria on HAZ

We carry out a meta-analysis in order to understand the variation in the estimates of the parameter of interest $\delta$, from all the 20 DHS. Let $\hat{\delta }_{k}$ and $s_{k}$ denote the maximum likelihood estimate of $\delta$ and its standard error, respectively, for $k=1,\ldots ,20$. We then model $\hat{\delta }_{k}$ using a weighted least squares fit to the regression model

3 $$\begin{aligned} \hat{\delta }_{k} = \alpha _{0} + \alpha _{1}v_{k} + Z_{k}, \end{aligned}$$

where $v_{k}$ is a World Bank African development indicator [41] associated with the country and year of the *k*-th survey, and the $Z_{k}$ are independent Gaussian variables with mean zero and variance $s_{k}^2$. We select eleven development indicators belonging to the categories of “Agriculture and rural development”, “Climate change”, “Economy and growth”, “Education” and “Environment”. A full list of the indicators is given in Additional file 2.

## Results

### Non-spatial analysis

**Fig. 1:**
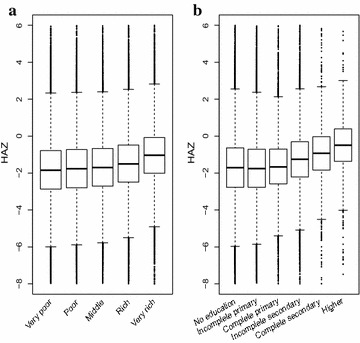
Box plots of height-for-age Z-scores by family’s wealth (**a**) and mother’s level of education (**b**), pooled over all 20 surveys

Figure 1 shows box-plots of HAZ by categories of family’s wealth indices and mother’s education level for all surveys combined. We assign integer scores 1–5 to the five levels of family wealth from very poor to very wealthy; and scores 1–6 to the six levels of mothers education, from no education to higher education. As expected, the box-plots show that the median HAZ tends to increase with increasing levels of wealth and education.

**Fig. 2:**
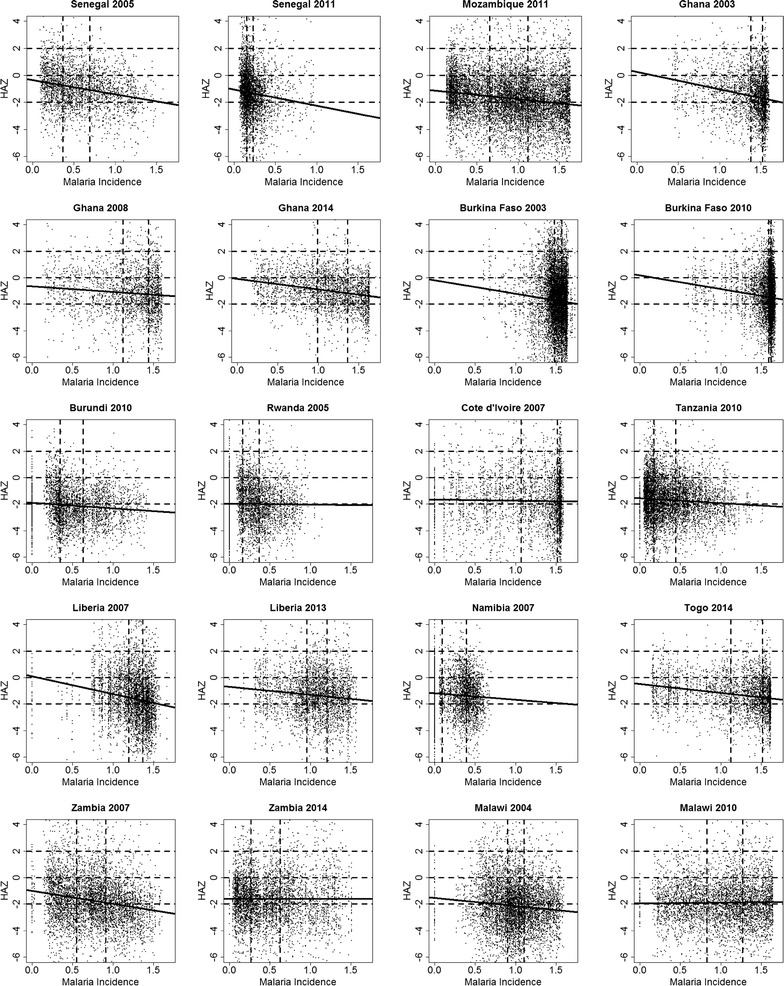
Scatterplots of height-for-age Z-scores (HAZ) against expected malaria incidence in the first year of life ($\mathcal {M}$). The solid line shows the univariate linear model with malaria incidence as the predictor of HAZ. The dashed horizontal lines show HAZ levels of 2, 0 and − 2, whilst the dashed horizontal lines separates $\mathcal {M}$ into terciles

**Fig. 3:**
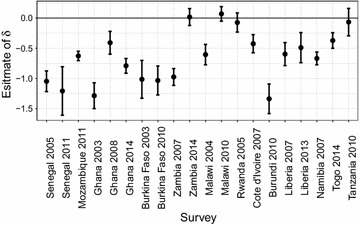
Plot of estimates of the malaria effect on HAZ with associated 95% confidence intervals obtained from a univariate linear model for each survey

We then investigate the marginal association between malaria incidence and HAZ. Figure 2 shows the observed HAZ against malaria incidence, where the solid line is obtained from the least squares fit of a univariate linear model. The dashed horizontal lines indicate HAZ levels of 2, 0 and − 2. The dashed vertical lines separate $\mathcal {M}$ into terciles. We see that Malaria incidence takes a maximum value of about 1.5 for all surveys, except Namibia in 2007, where this is about 0.7. We also note that for the surveys in Senegal in 2005, Mozambique in 2011, Ghana in 2003–2008–2014 and Zambia in 2007, the variation in $\mathcal {M}$ is evenly distributed, whereas it is more skewed for Senegal in 2011, Burkina Faso in 2003–2010, Malawi in 2004 and Namibia in 2007. Except for Rwanda in 2005, Zambia in 2014 and Malawi in 2010, in all the remaining 17 surveys we observe that HAZ decreases with increasing values of $\mathcal {M}$. Figure 3 shows the least squares estimates and the corresponding 95% confidence intervals. The estimated regression coefficients are negative in 18 surveys, of which 16 are significantly different from zero at 5% level.

**Fig. 4:**
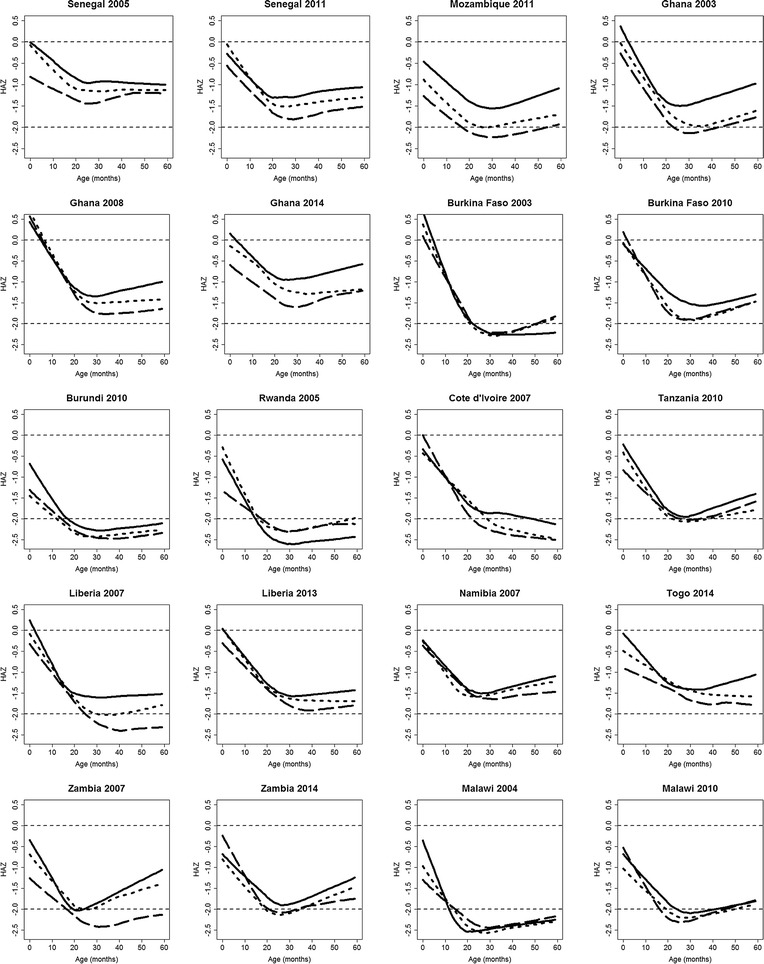
Estimated trajectories of height-for-age Z-scores (HAZ) as a function of age, stratified by malaria incidence ($\mathcal {M}$). Each panel shows three curves. Each curve is a piecewise cubic spline with knots at 12 and 24 months and corresponds to a tercile group of $\mathcal {M}$. The solid, dotted and dashed curves respectively correspond to the first, second and third terciles of $\mathcal {M}$, as indicated in Fig. 2. The horizontal lines are the HAZ levels of 0 and − 2

Figure 4 shows HAZ curves as functions of age, within each of the terciles groups of $\mathcal {M}$, as indicated in Fig. 2. The fitted curves reflect the typical age-related pattern of HAZ in LMICs: after a decrease in HAZ during the first 2 years of life, child-growth slowly recovers but never reaches zero. This phenomenon, known as “growth faltering”, has been widely observed; see, for example, [11, 12, 42, 43]. We also observe that in Burkina Faso in 2003, Ghana in 2008, Malawi in 2004–2010 and Rwanda in 2005, HAZ curves by terciles groups of $\mathcal {M}$ are partly overlapping, whereas in the remaining 15 surveys, children in the first tercile of $\mathcal {M}$ have the highest levels of HAZ and children in the third tercile with the lowest levels of HAZ, irrespective of age. We also notice that in Burkina Faso in 2003, Burundi in 2010, Rwanda in 2005, Cote d’Ivoire in 2007 and Malawi in 2004, where median HAZ curves fall below the − 2 threshold at about 24 months of age, the curves still remain below the − 2 threshold in later years.

### Geostatistical analysis

**Fig. 5:**
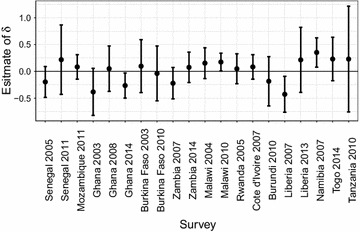
Plot of estimates of the malaria effect on HAZ with associated 95% confidence intervals, obtained from the geostatistical model in (1) for each survey

Figure 5 shows estimates, with associated $95\%$ confidence intervals, of the malaria parameter $\delta$ from the fitted geostatistical model in (1). The point estimate of $\delta$ is negative in 7 surveys with Ghana in 2014 and Liberia in 2007 being significant at the 5% level. Positive values are estimated for the remaining 13 surveys, with only Namibia in 2007 being significant. We note that, after accounting for residual spatial variation and measured potential confounders, the magnitude of the association between malaria incidence and HAZs is smaller than for the marginal association shown in Fig. 3.

Point estimates of the covariance parameters of (1) with associated standard errors are reported in Additional file 3. We see that, for each survey, the variance corresponding to the child-specific variation is consistently larger than both the variance of the spatial process and the nugget variance.

The results from the model validation (Additional file 4) show that the fitted geostatistical models are compatible with the data for each of the 20 surveys analysed. We also point out that, although the variograms based on the residuals from the standard linear regression are relatively flat, we still find evidence of non-negligible residual spatial variation in HAZ as indicated by the interval estimates of the parameter of the scale of the spatial correlation in Additional file 3.

### Mapping of stunting risk

**Fig. 6:**
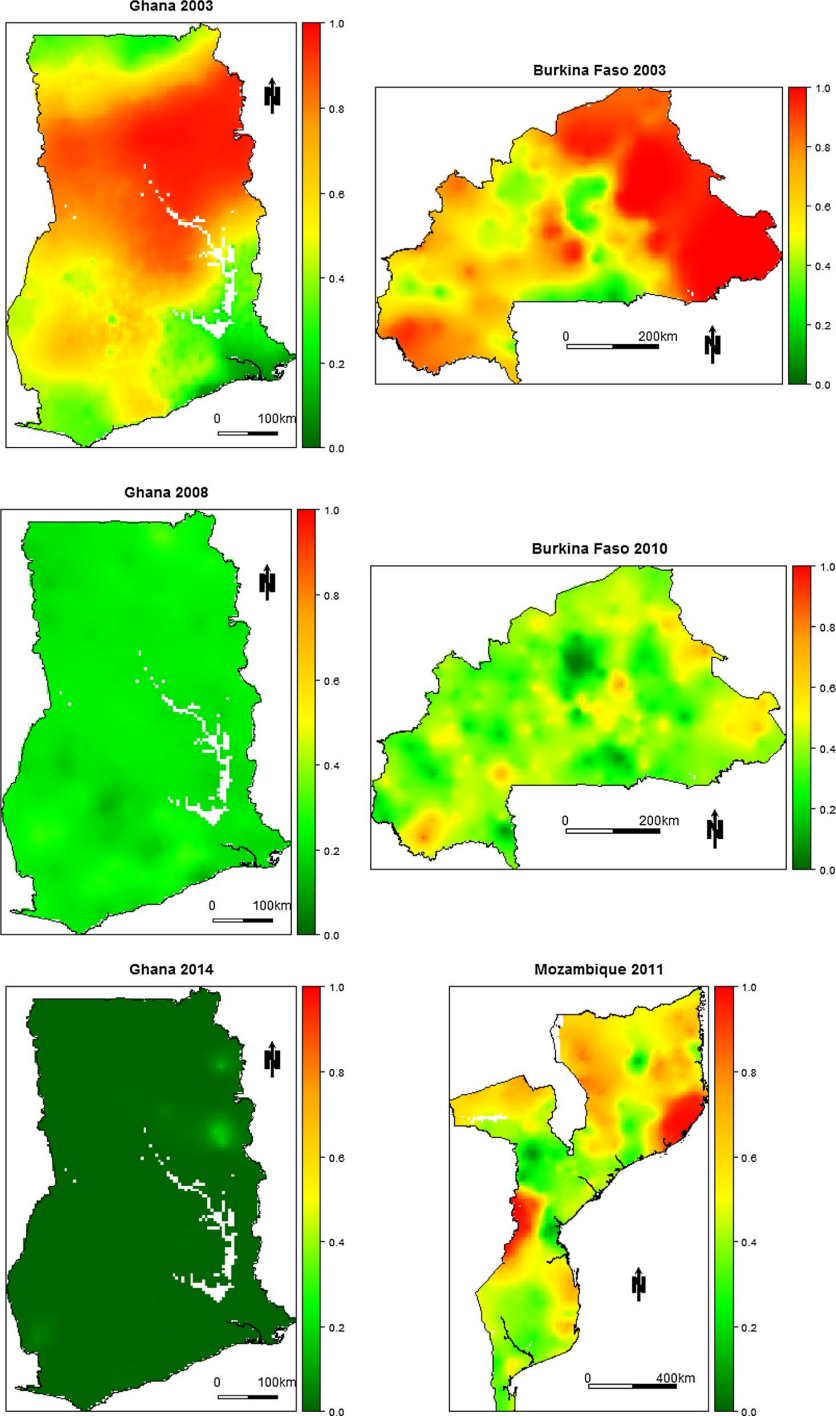
Predicted stunting risk maps for Ghana, Burkina Faso and Mozambique. The colour scale ranges from green to red with red areas being high risk areas and green areas being low risk areas

In Fig. 6, we report the predictive maps of stunting risk for Ghana, Burkina Faso and Mozambique for boys, aged 24 months. In Ghana in 2003–2008–2014, the maps show a remarkable decrease in stunting over time, that is observed almost everywhere within the country. Similarly, in Burkina Faso, we observe a decrease in stunting risk from 2003 to 2010. Mozambique in 2011 shows high spatial heterogeneity in stunting risk, with values ranging from 0.1 to 0.9. Risk maps for the remaining surveys are shown in Additional file 5. In these maps, we observe overall higher levels of stunting risk in Burundi in 2010 and Malawi in 2004, and lower levels in Senegal in 2008 and Togo in 2014.

### Variation in the effect of malaria on HAZ

The amount of arable land (defined as percentage of land under temporary crops, meadows for mowing or for pasture, market or kitchen gardens, and land temporarily fallow) in the country and year of survey is the only World Bank indicator to be significant at $5\%$ level, with a p-value of about 0.013, explaining 26% of the total variation in the estimated effects of malaria incidence on HAZ. More specifically, we estimate that an increase of 1% in arable land leads to a 0.008 increase in the value of the estimated malaria effect, on average. See Additional file 2 for more detailed results from the meta analysis.

## Discussion

The objective of our study was to model and quantify the association between malaria and HAZs in children aged less than 5 years. Using DHS data from 20 surveys in 13 African countries between 2003 and 2014, we have developed a geostatistical framework to model HAZ as a function of both child-specific and spatial risk factors. As a proxy for malaria exposure, we used estimates of malaria incidence in the first year of life from the Malaria Atlas Project. A non-spatial univariate linear regression showed a negative effect of malaria incidence on HAZs. However, after controlling for confounding and residual spatial effects, the estimated effect of malaria on HAZ was weaker and not significant in 17 out of the 20 surveys considered.

One of the main challenges in modelling the association between malaria and HAZ is the need to take account of confounding effects. Among these, socio-economic status has been shown to be one of the most important [44–47]. Education is another important factor that affects both malaria exposure and risk of stunting [34, 48, 49]. Higher levels of education are associated with improved knowledge and practice about the appropriate strategies for the prevention and treatment of malaria [50], and about healthy practices in breastfeeding and child nutrition [51]. Our results are consistent with these findings in all of the 20 surveys here analysed.

We observed that in surveys where HAZ curves fall below the − 2 threshold in early childhood, the curves never really rise above the − 2 threshold in later years. This finding suggests that recovery to standard growth after 2 years of age may be more difficult when the decrease in HAZ in early childhood is severe. This is consistent with the findings from [52] who showed that recovery from stunting is associated with the severity of stunting in early years. Other factors that have been found to favour recovery from low HAZ are good nutrition [53] and higher levels of mother’s education [54].

In our analysis, we found a mix of positive and negative point estimates of the association between malaria incidence and HAZ among the different surveys. However, findings from previous studies have shown contrasting results, with some reporting statistically significant negative associations between malaria and stunting [26, 29, 55, 56], and others reporting positive associations [30, 31]. To understand such variation in the magnitude and direction of the estimated parameters that quantify the malaria effect, we carried out a meta-analysis by considering several indicators of national development from the World Bank. Among these, the amount of arable land was the only one to show a significant association. Arable land might in fact modulate the association between malaria and HAZ, with a larger surface of arable land leading to a fall in poverty and malnutrition, especially in rural areas [57], but also to a larger number of breeding sites for mosquitoes [58]. This suggests that geo-political differences among countries should also be considered, since the implementation of policies aiming to reduce malnutrition can also impact on the epidemiology of malaria. Arable land could be indeed associated with agricultural, economic and environmental factors that are common to both malaria and stunting [59, 60].

We have quantified stunting risk by mapping the predictive probability that HAZ is below a threshold of − 2. For countries with repeated surveys, our risk maps showed reductions over time in the risk of stunting. The main factors that might be driving such reductions are improvements in health environments through increasing access to safe water and sanitation, improvements in the quality of caring practices for children through increasing women’s education and promoting gender equality, including women’s empowerment; and increase in food security by ensuring adequate availability of food at the national level and sufficient nutritional quality of that food [59, 61, 62]. Our risk maps showed remarkable spatial heterogeneity in the risk of stunting, identifying geographic areas with high risk that could be considered for a more targeted intervention.

It has been widely observed that HAZ undergoes a rapid decrease in the first 24 months and an increase thereafter [11, 12, 42]. For this reason we used cubic splines with knots at 12 and 24 months in order to better capture the non-linear trajectory that we observed across the 5 years of age.

### Limitations of the study

The main limitation of our study is that the information available to us on malaria and HAZ is cross-sectional, rather than longitudinal, in nature. This prevents us from establishing whether our observed associations can be given a causal interpretation. A second limitation is that we have no information on the uncertainty associated with the estimates of malaria incidence. We have assumed the first year of life to be the most important in determining the strength of the association between malaria and child growth. To investigate whether exposure to malaria in other years of childhood could also have an impact on growth would require the fitting of a distributed lag-model.

In Additional file 6, we give methodological details on how to account for uncertainty in malaria incidence in a cross-sectional geostatistical setting.

To assess the cumulative effect of malaria on child-growth at different developmental stages, we would need longitudinal, individual-level data on children’s actual malaria status over the first 5 years of life. We would then extend our current methodology as follows.

#### Novel extensions to longitudinal geostatistical data

To simplify the notation and without loss of generality, we assume that all the sampled children have identical follow up times. Then, let $Y_{ijt}$ and $W_{ijt}$ denote the HAZ and number of malaria episodes for the *j*-th child at location $x_i$ and time *t*, respectively. Also, let $\tilde{S}(x, t)$ denote a latent spatio-temporal Gaussian process. Given $\tilde{S}(x, t)$, we model the $W_{ijt}$ as a set of mutually independent Poisson variables with mean ${\mathcal {M}}_{ijt}$ such that

$$\begin{aligned} \log \{{\mathcal {M}}_{ijt}\} = \tilde{e}_{ijt}^\top \tilde{\gamma } + \tilde{d}(x_{i})^\top \tilde{\beta } + \tilde{S}(x_i, t), \end{aligned}$$

where $\tilde{e}_{ijt}$ are child-specific explanatory variables that might vary over time. We then assume that $Y_{ijt}$, conditionally on ${\mathcal {M}}_{ijt}$, a spatio-temporal Gaussian process *S*(*x*, *t*) and random effects $U_{it}$ and $V_{ij}$, are independent Gaussian variables with mean

4 $$\begin{aligned} \mu _{j}(x_i, t) = e_{ijt}^\top \gamma + d(x_i, t) \beta + \sum _{h=0}^{t-1}\delta _{t-h} {\mathcal {M}}_{ij(t-h)} + f({\mathcal {A}}_{ijt}) + V_{ij} + S(x_i, t) + U_{it} \end{aligned}$$

In (4), $U_{it}$ is unstructured unexplained variation at location $x_{i}$ and time *t*, $V_{ij}$ is unexplained child-specific variation and the lagged parameters $\delta _{t-h}$, for $h=0,\ldots ,t=1$, represents the effect of malaria incidence during the *h*-th year of life on HAZ. To make the model more parsimonious, the parameters $\delta _{t-h}$ can be constrained using a parametric specification, i.e. $\delta _{t-h} = g(t-h; \theta )$ where $g(\cdot ; \theta )$ is a known function indexed by the vector of parameters $\theta$.

This modelling framework would allow us to better understand the cumulative effect of malaria on HAZ at different developmental stages by overcoming the current limitation of our study where we assume that $\delta _{t-h}=0$ for $0\le h \le t-2$.

## Conclusion

Geostatistical methods provide a useful framework to account for spatially structured confounding effects that modulate the association between malaria and HAZ. This study also highlights that one of the main challenges in modelling this association is that confounding effects vary by country, as well as in time. This can change both the direction and magnitude of the effect of malaria on HAZ, making a generalization on the effect of malaria on HAZ almost impossible using only currently available data. Establishing whether the association between malaria and stunting is causal would require longitudinal follow-up data on individual children.

## Additional files

**Additional file 1.** Computational details.

**Additional file 2.** Details of the World Bank development indicators.

**Additional file 3.** Estimates of covariance parameters.

**Additional file 4.** Results from the model validation.

**Additional file 5.** Maps of stunting risk.

**Additional file 6.** Accounting for the uncertainty in malariaincidence.

## Abbreviations

DHS: Demographic and Health Surveys
GDP: gross domestic product
HAZs: height-for-age Z-scores
LMICs: low- and middle-income countries
WHO: World Health Organization
