# Supplementary material for: Geostatistical modelling of the association between malaria and child growth in Africa
Source: Int J Health Geogr. 2018 Feb 27;17:7. doi: 10.1186/s12942-018-0127-y (PMC5828493; doi:10.1186/s12942-018-0127-y)
Supplement: Supplementary file 1 — Additional file 1. Computational details. [file 12942_2018_127_MOESM1_ESM.pdf]

Additional file 1: Computational details

Parameter estimation

Let $Y^\top = (Y_1^\top, \dots, Y_n^\top)$, where Y_i is the vector of observed HAZs at the ith cluster, and Let $N = \sum_{i=1}^n m_i$. The vector Y has a multivariate Gaussian distribution with mean $\mu = D\xi$ and covariance matrix Σ, where D is a matrix of covariates including the cubic spline bases, with vector of regression coefficients $\xi^\top = (\gamma^{*\top}, \beta, \delta)$, where γ^* consists of coefficients of child-specific variables, spatial variables and those of the spline bases, and $\Sigma = C\Big(\sigma^2 R + \tau^2 I_n\Big)C^\top + \omega^2 I_N$, where $[R]_{ij} = \rho(u_{ij}; \phi)$ and

$$[C]_{ij} = \begin{cases} 1 & \text{if the } j^{th} \text{ child has been sampled at location } x_i, \\ 0 & \text{otherwise} \end{cases}.$$

Let $\theta^\top = (\xi^\top, \sigma^2, \tau^2, \omega^2, \phi)$ denote the vector of model parameters; the log-likelihood for θ is given by

$$L(\theta) = -\frac{1}{2}\Big\{ \log|\Sigma| + (y-\mu)^T \Sigma^{-1}(y-\mu)\Big\}. \quad \text{(AF1.1)}$$

Inversion of the covariance matrix Σ can be simplified using the Woodbury matrix identity to give

$$\Sigma^{-1} = (\tilde{\omega}^{-2} I_N - \tilde{\omega}^{-4} C\Big[(R + \tilde{\tau}^2 I_n)^{-1} + \tilde{\tau}^{-2} C^T C\Big]^{-1} C^T)/\sigma^2. \quad \text{(AF1.2)}$$

where $\tilde{\omega}^2 = \omega^2/\sigma^2$ and $\tilde{\tau}^2 = \tau^2/\sigma^2$. Using Sylvester's determinant identity, we can also write

$$|\Sigma| = \omega^{2N} |\tilde{\omega}^{-2} C^\top C(R + \tilde{\tau}^2 I_n) + I_n|, \quad \text{(AF1.3)}$$

hence, as in (AF1.2), computations are carried out on an n by n matrix.

To maximize $L(\theta)$, we then use the profile likelihood for given $\psi = (\tilde{\tau}^2, \tilde{\omega}^2, \phi)$. The profile estimates for ξ and σ^2 are respectively given by

$$\hat{\xi}(\psi) = (D^T Q D)^{-1} D^T Q^{-1} y$$

and

$$\hat{\sigma}^2(\psi) = \frac{1}{N}(y - D\hat{\xi}(\psi))^T Q^{-1}(y - D\hat{\xi}(\psi))$$

where $\sigma^2 Q = \Sigma$. By plugging $\hat{\xi}(\theta)$ and $\hat{\sigma}^2(\theta)$ into (AF1.1), we obtain

$$L_p(\psi) = -\frac{1}{2}\left\{N \log \hat{\sigma}^2(\psi) + \log |Q|\right\}. \qquad \text{(AF1.4)}$$

Finally, numerical optimization can be used to maximize $L_p(\psi)$ with respect to ψ.

Model validation

We carry out model validation to test the validity of the adopted spatial covariance function as follows.

Let $W_j(x_i) = S(x_i) + U_i + V_i$ denote the residual variation in HAZ for j-th children at location x_i, where $V_i \sim \mathcal{N}(0, \omega^2)$ and $S(x_i)$ and $U(x_i)$ are as defined in the geostatistical model for HAZ in equation (1) of the manuscript. The theoretical variogram of the random effects is

$$\gamma(u_{hk}) = \omega^2 + \tau^2 + \sigma^2(1 - \exp\{u_{hk}\}) \qquad \text{(AF1.5)}$$

where u_{hk} is the Euclidean distance between location x_h and x_k.

Denote by $\tilde{W}_j(x_i)$ the estimated residuals from a standard linear regression for the j-th child at location x_i. Let $N(u) = \{(h,k) : ||x_h - x_k|| = u_{hk}\}$, i.e. the set of all data-points such

that their distance is u_{hk}. The empirical variogram is the defined as

$$\tilde{\gamma}(u_{hk}) = \frac{1}{2|N(u_{hk})|} \sum_{(h,k)\in N(u)} \left(\tilde{W}_j(x_h) - \tilde{W}_{j'}(x_k)\right)^2, \tag{AF1.6}$$

where $|N(u_{hk})|$ is the number of observations in $N(u_{hk})$.

To generate a 95% bandwidth of the empirical variogram under the fitted model, we first simulate $W_j(x_i)$, at observed locations x_i, from its marginal multivariate Gaussian distribution, as defined by the geostatistical model. Conditionally on the simulated values of $W_j(x_i)$, we simulate HAZ from the conditional model in equation (1) of the manuscript. We then compute the empirical variogram in (AF1.6) obtained from the simulated data. We repeat this process 1,000 times. Finally, we generate 95% tolerance intervals at each of pre-defined spatial distances of the variogram.

Spatial prediction

Let $T^\top = (T(x_1^*), \ldots, T(x_q^*))$ denote our target of prediction, where x_i^* are q prediction locations. The conditional distribution of T given the data $Y = y$ and all the explanatory variables at each of the prediction locations x_i^*, is a multivariate Gaussian with mean

$$D^*\xi + P\Sigma^{-1}(y - D\xi), \tag{AF1.7}$$

where D^* is the matrix of explanatory variables at the prediction locations, P is the cross-covariance matrix and ξ the vector of all the regression coefficients reported in equation (1) in the manuscript; the covariance matrix is

$$\sigma^2(R^* + \tilde{\tau}^2 I) - P\Sigma^{-1}P^\top, \tag{AF1.8}$$

where $[R^*]_{ij} = \exp\{-u_{ij}^*/\phi\}$ and u_{ij}^* is the Euclidean distance between any two prediction locations x_i^* and x_j^*. When carrying out predictions, we plug-in the maximum likelihood estimates for each of the model parameters.

In order to quantify the risk of stunting at a location x, we map

$$\text{Prob}(T(x_i^*) < -2|y), i = 1, \ldots, q. \tag{AF1.9}$$

In the above equation we fix age at 24 months and gender to male, whilst we integrate out maternal education and wealth index as follows. Let $[\cdot]$ be a shorthand notation for "the distribution of $\cdot$". The predictive distribution of the target $T(x)$ is then given by

$$[T(x_i^*)|y] = \int [\mathcal{D}][T(x_i^*)|y, \mathcal{D}]d\mathcal{D}, i = 1, \ldots, q \tag{AF1.10}$$

where $\mathcal{D} = (\mathcal{W}, \mathcal{E})$, with $\mathcal{E}$ corresponding to maternal education and $\mathcal{W}$ to wealth index, and $[T(x_i)|y, \mathcal{D}]$ is the i-th component of the multivariate Guassian distribution with mean and covariance matrix given by (AF1.7) and (AF1.8), respectively. We model the joint distribution of $\mathcal{D}$ as

$$[\mathcal{D}] = [\mathcal{E}][\mathcal{W}|\mathcal{E}]$$

where $[\mathcal{E}]$ is estimated using the empirical distribution obtained from the data of a given survey, and $[\mathcal{W}|\mathcal{E}]$ is a proportional odds cumulative probit model [1].

To compute (AF1.10), we then generate 10,000 samples from $[T(x_i^*)|y]$ by simulating sequentially from $[\mathcal{E}]$, $[\mathcal{W}|\mathcal{E}]$ and $[T(x_i^*)|y, \mathcal{D}]$. Finally, we obtain (AF1.9) by computing the proportions of simulated samples from $[T(x_i^*)|y]$ that lie below -2, for $i = 1, \ldots, q$.

References

[1] Agresti, A.: Categorical Data Analysis vol. 990. John Wiley & Sons, New York (1996)
