# Supplementary material for: Geostatistical modelling of the association between malaria and child growth in Africa
Source: Int J Health Geogr. 2018 Feb 27;17:7. doi: 10.1186/s12942-018-0127-y (PMC5828493; doi:10.1186/s12942-018-0127-y)
Supplement: Supplementary file 2 — Additional file 2. Details of the World Bank development indicators. [file 12942_2018_127_MOESM2_ESM.pdf]

Additional file 2: Details of the World Bank development indicators

Additional Table 1. World Bank Development Indicators.

Indicator	Description
Land under cereal production	Land under cereal production refers to harvested area, although some countries report only sown or cultivated area. Cereals include wheat, rice, maize, barley, oats, rye, millet, sorghum, buckwheat, and mixed grains.
Arable land	Arable land includes land defined as land under temporary crops (double-cropped areas are counted once).
Livestock production index	Livestock production index includes meat and milk from all sources, dairy products such as cheese, and eggs, honey, raw silk, wool, and hides and skins.
GDP per capita	GDP per capita based on purchasing power parity.
Primary completion rate, male	Primary completion rate is the number of new entrants in the last grade of primary education, regardless of age, divided by the population at the entrance age for the last grade of primary education.
Population density	Population density is midyear population divided by land area in square kilometers.
Progression to secondary school, female	Progression to secondary school refers to the number of new entrants to the first grade of secondary school in a given year as a percentage of the number of students enrolled in the final grade of primary school in the previous year (minus the number of repeaters from the last grade of primary education in the given year).
Prevalence of anemia among children	Prevalence of anemia, children under age 5, is the percentage of children under age 5 whose hemoglobin level is less than 110 grams per liter at sea level.
Improved water source	Access to an improved water source refers to the percentage of the population using an improved drinking water sources which include piped water on premises and other improved drinking water sources such as public taps or standpipes, tube wells or boreholes, protected dug wells, protected springs, and rainwater collection.
Improved sanitation facilities, rural/urban	Access to improved sanitation facilities refers to the percentage of the population using improved sanitation facilities. Improved sanitation facilities include flush/pour flush (to piped sewer system, septic tank, pit latrine), ventilated improved pit (VIP) latrine, pit latrine with slab, and composting toilet.
Unemployment	Unemployment refers to the share of the labor force that is without work but available for and seeking employment.

Additional Table 2. Regression table for the meta analysis

World Bank Development Indicator	Point Estimate	Standard Error	p-value
Land under cereal production	5.0388×10^{-8}	4.3233×10^{-8}	0.2590
Arable land	7.9548×10^{-3} *	2.8915×10^{-3}	0.0131
Livestock production index	5.7663×10^{-4}	1.6313×10^{-3}	0.7278
GDP per capita	-4.1747×10^{-5}	3.5107×10^{-5}	0.2498
Primary completion rate, male	-2.3216×10^{-3}	2.9019×10^{-3}	0.4341
Population density	5.5419×10^{-4}	4.8843×10^{-4}	0.2714
Progression to secondary school, female	-3.8597×10^{-3}	3.6859×10^{-3}	0.3089
Prevalence of anaemia among children	-4.6101×10^{-3}	4.7033×10^{-3}	0.3400
Improved water source	-5.1427×10^{-3}	4.3177×10^{-3}	0.2491
Improved sanitation facilities, urban	2.7747×10^{-3}	3.5442×10^{-3}	0.4439
Unemployment	5.0836×10^{-3}	8.4332×10^{-3}	0.5542

Dependent Variable: Estimate of malaria-HAZ association parameter
Significance levels: 0 '***' 0.001 '**' 0.01 '*' 0.05 '.' 0.1 ' ' 1
