# Supplementary material for: Geostatistical modelling of the association between malaria and child growth in Africa
Source: Int J Health Geogr. 2018 Feb 27;17:7. doi: 10.1186/s12942-018-0127-y (PMC5828493; doi:10.1186/s12942-018-0127-y)
Supplement: Supplementary file 3 — Additional file 3. Estimates of covariance parameters. [file 12942_2018_127_MOESM3_ESM.pdf]

Additional file 3: Estimates of covariance parameters

Additional Table 3. Estimates of Covariance Parameters

Survey	$\log(\sigma^2)$	$\log(\phi)$	$\log(\tau^2)$	$\log(\omega^2)$
Senegal 2005	-4.130 (0.981)	4.520 (0.983)	-2.055 (2.042)	0.659 (1.963)
Senegal 2011	-2.915 (0.522)	4.311 (0.724)	-2.180 (1.171)	0.951 (1.045)
Mozambique 2011	-1.802 (0.333)	5.001 (0.457)	-2.197 (0.754)	0.959 (0.666)
Ghana 2003	-2.388 (0.493)	4.780 (0.705)	-2.565 (1.136)	0.687 (0.987)
Ghana 2008	-2.995 (1.552)	3.056 (1.488)	-1.290 (3.355)	0.930 (3.105)
Ghana 2014	-3.154 (0.494)	4.551 (0.786)	-3.601 (1.407)	0.323 (0.988)
Burkina Faso 2003	-0.696 (0.530)	5.328 (0.693)	-2.289 (1.068)	1.105 (1.059)
Burkina Faso 2010	-2.426 (0.394)	3.500 (0.503)	-1.981 (0.971)	0.833 (0.789)
Zambia 2007	-1.886 (0.651)	6.025 (0.854)	-2.755 (1.377)	1.115 (1.303)
Zambia 2014	-2.713 (0.487)	3.600 (1.124)	-1.830 (1.138)	1.041 (0.975)
Malawi 2004	-2.905 (0.674)	4.975 (1.225)	-2.678 (1.441)	1.117 (1.348)
Malawi 2010	-3.329 (0.682)	3.086 (0.634)	-2.443 (1.628)	1.015 (1.365)
Rwanda 2005	-3.0599 (0.549)	3.019 (0.731)	-1.970 (1.221)	0.812 (1.099)
Cote d'Ivoire 2007	-1.155 (0.678)	1.798 (0.869)	-2.186 (3.046)	1.375 (1.357)
Burundi 2010	-2.909 (0.424)	2.122 (0.527)	-2.816 (1.175)	0.502 (0.848)
Liberia 2007	-2.885 (0.745)	2.955 (0.648)	-2.665 (1.966)	1.111 (1.491)
Liberia 2013	-3.324 (0.700)	3.563 (0.693)	-2.821 (1.741)	0.874 (1.400)
Namibia 2007	-2.255 (0.431)	5.011 (0.522)	-3.714 (1.447)	0.743 (0.862)
Togo 2014	-3.335 (1.454)	1.674 (0.714)	-3.847 (5.168)	0.540 (2.908)
Tanzania 2010	-2.313 (0.304)	4.860 (0.489)	-3.175 (0.853)	0.670 (0.607)

Standard errors in brackets
