# Supplementary material for: Geostatistical modelling of the association between malaria and child growth in Africa
Source: Int J Health Geogr. 2018 Feb 27;17:7. doi: 10.1186/s12942-018-0127-y (PMC5828493; doi:10.1186/s12942-018-0127-y)
Supplement: Supplementary file 4 — Additional file 4. Results from the model validation. [file 12942_2018_127_MOESM4_ESM.pdf]

Additional file 4: Results from the model validation

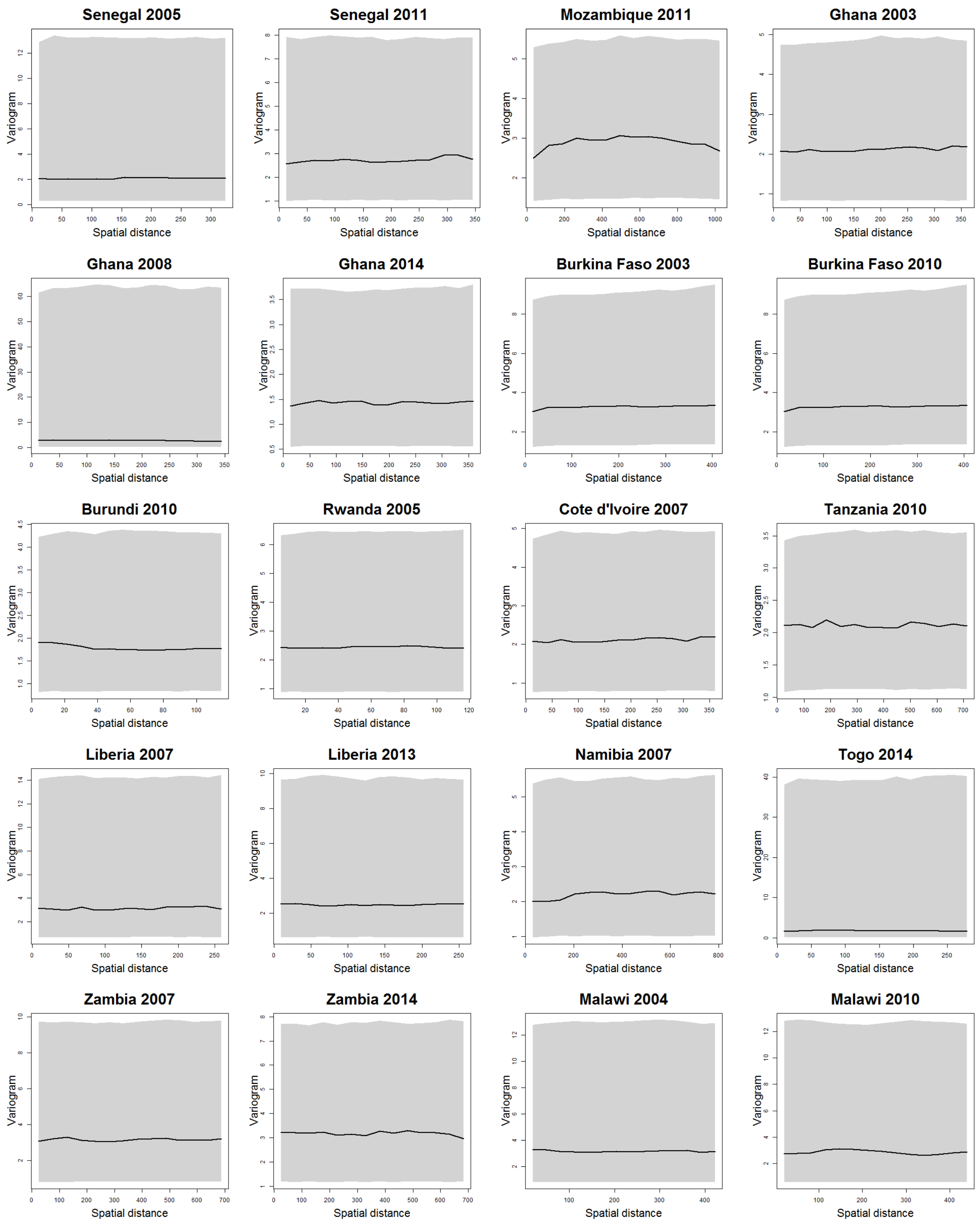

Additional Figure 1. The solid line corresponds the empirical variogram of the residuals from a standard linear regression analysis. The shaded area is the 95% tolerance bandwidth generated under the hypothesis that the adopted correlation function is the true model.
