# Supplementary material for: Geostatistical modelling of the association between malaria and child growth in Africa
Source: Int J Health Geogr. 2018 Feb 27;17:7. doi: 10.1186/s12942-018-0127-y (PMC5828493; doi:10.1186/s12942-018-0127-y)
Supplement: Supplementary file 5 — Additional file 5. Maps of stunting risk. [file 12942_2018_127_MOESM5_ESM.pdf]

Additional file 5: Maps of stunting risk

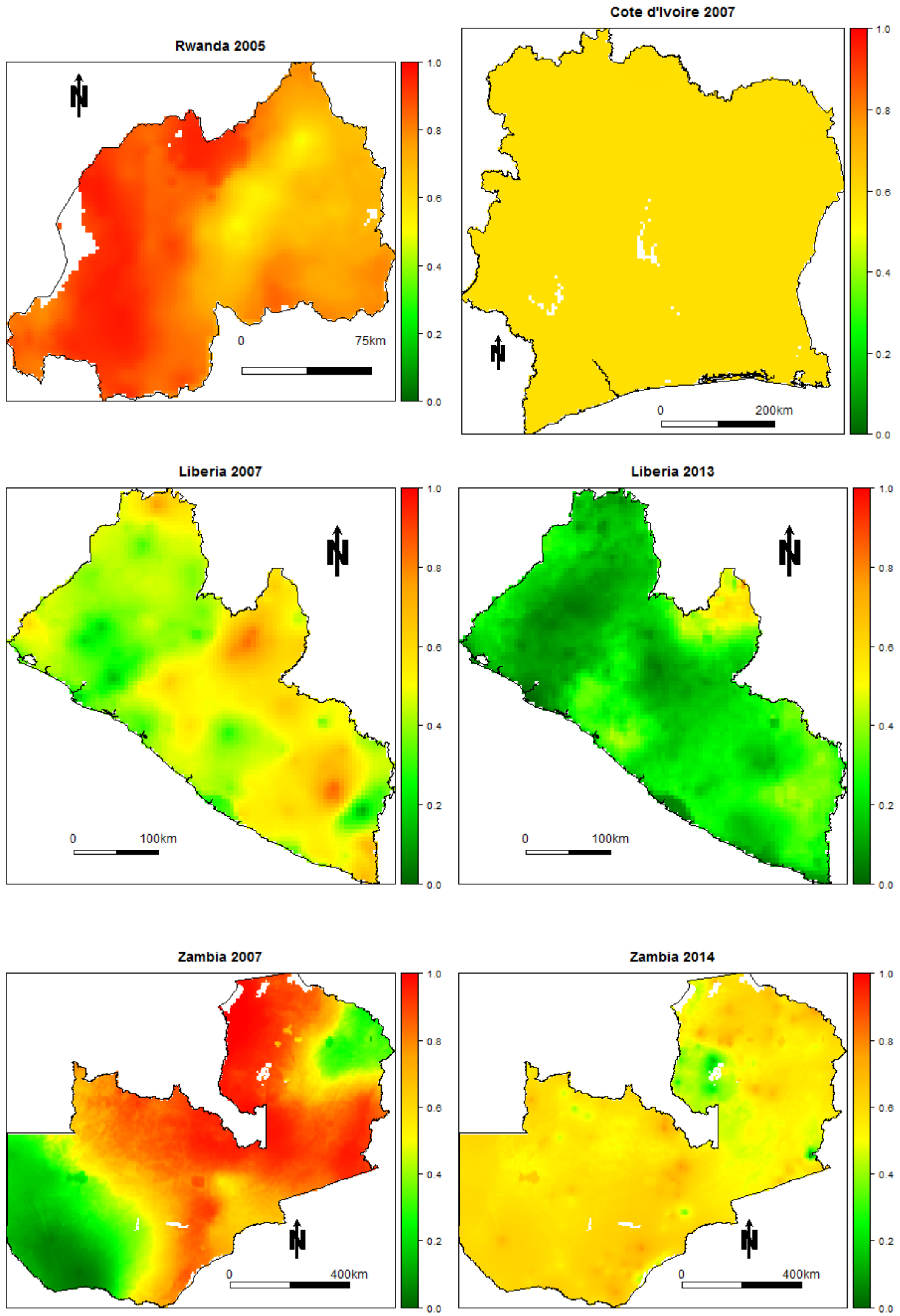

Additional Figure 2. Predicted stunting risk maps for Rwanda, Cote d'voire, Liberia and Zambia.

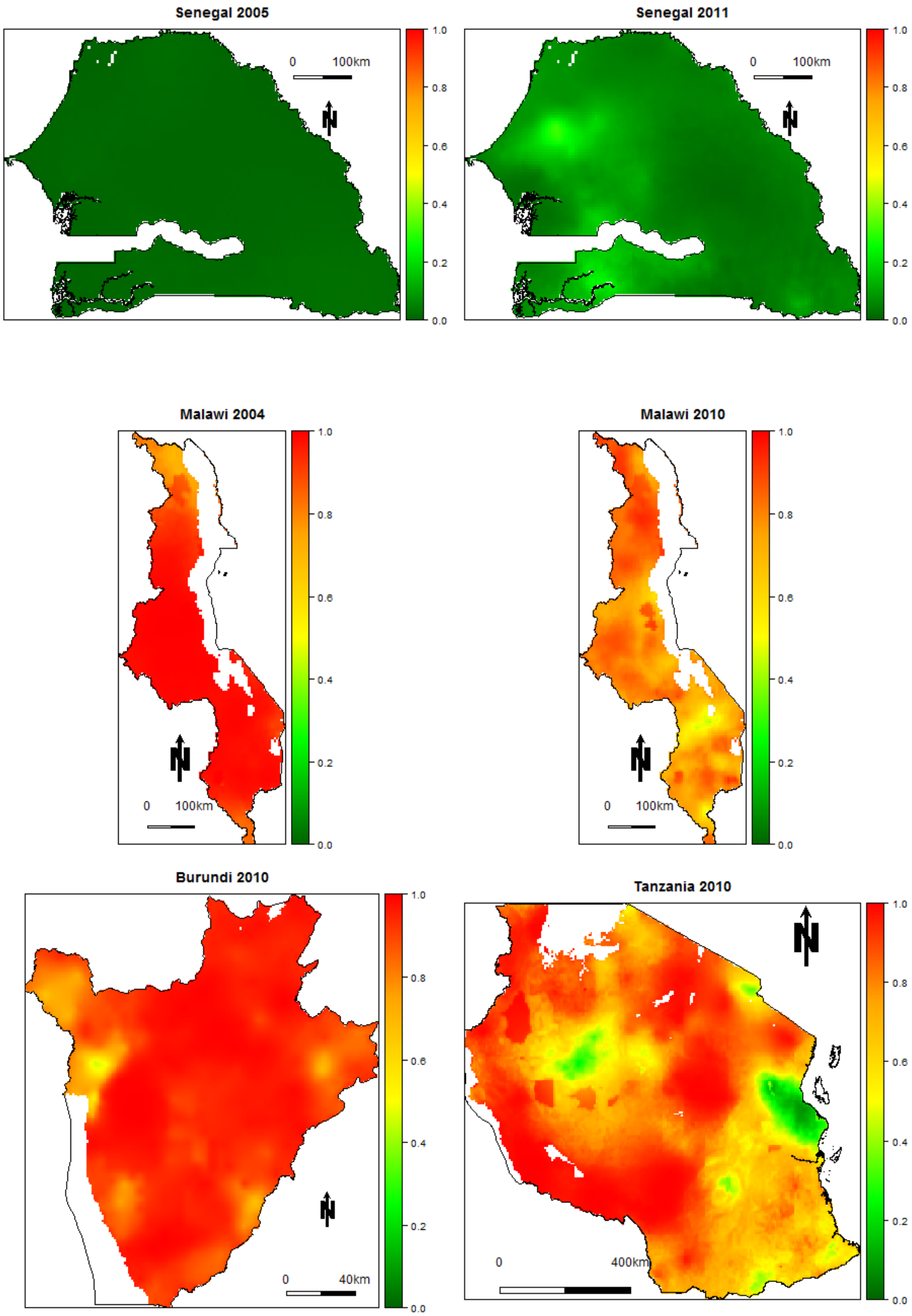

Additional Figure 3. Predicted stunting risk maps for Senegal, Malawi, Burundi and Tanzania.

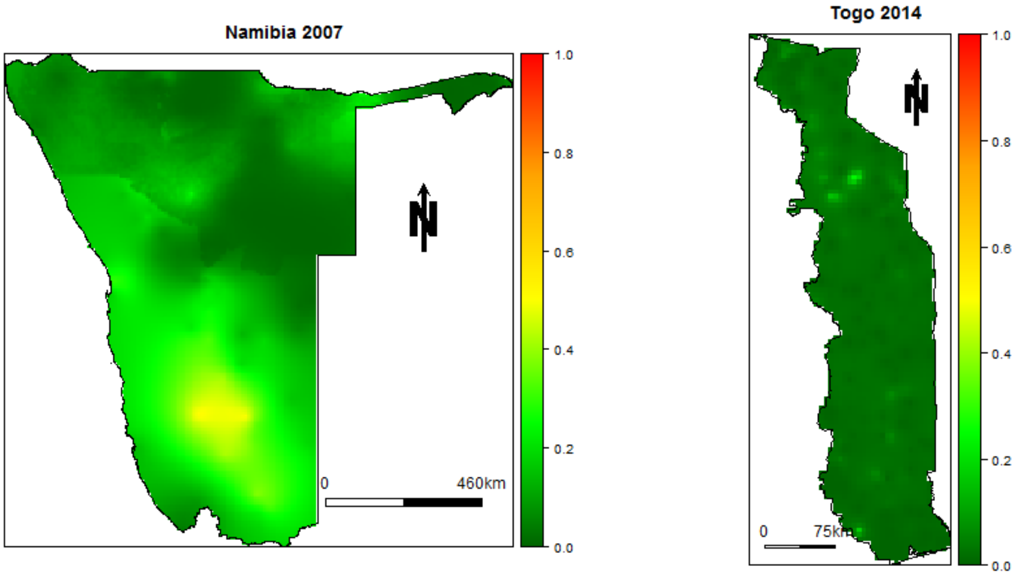

Additional Figure 4. Predicted stunting risk maps for Namibia and Togo.
