# Supplementary material for: Geostatistical modelling of the association between malaria and child growth in Africa
Source: Int J Health Geogr. 2018 Feb 27;17:7. doi: 10.1186/s12942-018-0127-y (PMC5828493; doi:10.1186/s12942-018-0127-y)
Supplement: Supplementary file 6 — Additional file 6. Accounting for the uncertainty in malariaincidence. [file 12942_2018_127_MOESM6_ESM.pdf]

Additional file 6: Accounting for the uncertainty in malaria incidence

To account for the uncertainty in malaria incidence, we consider two possible scenarios: (A) predictive samples or measures of uncertainty from the model used to estimate malaria incidence are available; (B) the data on malaria incidence are available. In what follows, we shall use $\mathcal{M}_{ij}$ to denote the mean of the predictive distribution of malaria incidence during the first year of life of the j-th child at location x_i.

Scenario (A). Let $\mathcal{M}_{ij}^{(r)}; r = 1, 2, \ldots, R$ be predictive samples of malaria incidence for the j-th child at location x_i. The resulting likelihood function is now obtained by averaging over the samples $\mathcal{M}_{ij}^{(r)}$, i.e.

$$\frac{1}{R}\sum_{r=1}^{R}[Y_{ij}|\mathcal{M}_{ij}^{(r)}] = \frac{1}{R}\sum_{r=1}^{R}\int\int[S(x_i)][U_i][Y_{ij}|S(x_i), U_i, \mathcal{M}_{ij}^{(r)}]\,dU_i\,dS(x_i), \qquad \text{(AF6.1)}$$

where $[\cdot]$ is a shorthand notation for "the distribution of $\cdot$" and $[Y_{ij}|S(x_i), U_i, \mathcal{M}_{ij}^{(r)}]$ is a Gaussian distribution with mean as in equation (1) of the main manuscript and variance ω^2. The expression for $[Y_{ij}|\mathcal{M}_{ij}^{(r)}]$ is given in "Additional file 1". The resulting estimate of δ and its standard error based on (AF6.1) incorporate the uncertainty in $\mathcal{M}_{ij}$.

However, due to limited computer memory, the predictive samples $\mathcal{M}_{ij}^{(r)}$ may not have been stored but summaries of the overall dispersion, such as standard errors, might instead be available. Let v_{ij}^2 denote the variance of the predictive distribution for malaria incidence. By approximating this with a log-Gaussian distribution, we then generate samples $\mathcal{M}_{ij}^{(r)}$ on the logarithmic scale by simulating from a Gaussian distribution with mean

$$\log\left\{\frac{\mathcal{M}_{ij}}{\sqrt{1+\frac{v_{ij}^2}{\mathcal{M}_{ij}}}}\right\}$$

and variance

$$\log\left\{1+\frac{v_{ij}^2}{\mathcal{M}_{ij}^2}\right\}.$$

Finally, we can use the resulting samples $\mathcal{M}_{ij}^{(r)}$ as in (AF6.1). However, the validity of this approach largely depends on the accuracy of the log-Gaussian approximation which is not feasible in cases where $\mathcal{M}_{ij} = 0$.

Scenario (B). In this scenario, the availability of data on malaria would allow us to develop a bivariate model for HAZ and the number of malaria episodes, denoted by W_{ij}, experienced by a child during his first year of life. This is preferred to *Scenario A* because we can then model the underlying process of malaria incidence within a geostatistical framework that is consistent with our approach used for HAZ. More specifically, we would assume that W_{ij}, conditionally on a spatial Gaussian process $\tilde{S}(x_i)$, are mutually independent Poisson variables, such that

$$\log\{\mathcal{M}_{ij}\} = \tilde{e}_{ij}^{\top}\tilde{\gamma} + \tilde{d}(x_i)^{\top}\tilde{\beta} + \tilde{S}(x_i), \qquad \text{(AF6.2)}$$

where $\tilde{e}_{ij}$ and $\tilde{d}(x_i)$ are child-specific and spatially referenced explanatory variables with associated regression coefficients $\tilde{\gamma}$ and $\tilde{\beta}$, respectively. The joint likelihood for Y_{ij} and W_{ij} is then given by

$$[Y_{ij}, W_{ij}] = \int\int\int [S(x_i)][U_i][\mathcal{M}_{ij}][Y_{ij}|S(x_i), U_i, \mathcal{M}_{ij}][W_{ij}|\mathcal{M}_{ij}]\, dU_i\, dS(x_i)\, d\mathcal{M}_{ij}. \quad \text{(AF6.3)}$$

Monte Carlo methods could then be used to approximate the above integral which is not available in closed form; see, for example, [1].

References

[1] Christensen, O.F.: Monte carlo maximum likelihood in model-based geostatistics. Journal of Computational and Graphical Statistics **13**(3), 702–718 (2004)
